# Accuracy of breathing and radial pulse assessment by non-medical persons: an observational cross-sectional study

**DOI:** 10.1038/s41598-023-28408-3

**Published:** 2023-01-31

**Authors:** Kensuke Suzuki, Ryoto Sakaniwa, Nobuko Endo, Miho Kubota, Mayumi Nakazawa, Kenji Narikawa, Satoo Ogawa, Hiroyuki Yokota

**Affiliations:** 1 The Graduate School of Health and Sport Science, Nippon Sport Science University, 1221-1 Kamoshida-Cho, Aoba-Ku, Yokohama City, Kanagawa 227-0033 Japan; 2 Department of Social Medicine, Osaka University Graduate School of Medicine, Suita, Osaka Japan; 3 Department of Health and Nutrition, Undergraduate School of Nutrition Sciences, Kagawa Nutrition University, Sakado, Saitama Japan

**Keywords:** Respiratory signs and symptoms, Preventive medicine

## Abstract

Early recognition of cardiopulmonary arrest (CPA) expedites emergency calls and resuscitation and improves the survival rate of unresponsive individuals. However, the accuracy of breathing and radial artery pulse assessment by non-medical persons is poorly understood. The aim of this study was to determine the accuracy of breathing assessment and radial pulse palpation among 450 non-medical personnel using a high-fidelity simulator. We examined the accuracy of 10 second’s assessment for breathing and radial pulse using a high-fidelity mannequin simulator, included 496 non-medical participants (school teachers) between 2016–2018. For a primary results, the sensitivity for the detection of the presence of the breathing and radial pulse was 96.2% (97.5% for sensitivity and 92.0% for specificity) and 91.7% (99.1% for sensitivity and 56.8% for specificity), respectively. Futher, breathing rate and radial pulse rate were strongly correlated with the assessments, with Spearman’s correlation coefficients of 0.813 (P < 0.001) and 0.719 (P < 0.001), respectively. In contrast, radial pulse strength was weakly correlated with the assessment (coefficient of 0.288, P < 0.001). Our results suggested that non-medical persons would show high accuracy in detecting and measuring respiration and radial pulse, although they did not accurately determine radial pulse strength for the early recognition of CPA.

## Introduction

Early recognition of cardiopulmonary arrest (CPA) can prevent delays in emergency calls and starting resuscitation, possibly improving the survival of an unresponsive person^1,2^. Early and clear assessment of breathing and pulse outside the hospital, at home, or in public places and nursing homes is associated with better survival and neurological disability^3^. However, according to a report from the Tokyo Fire Department, Japan, in 2019, approximately 75% of the bystanders were not medical practitioners^4^. Thus, the situation such as a recognition of CPA might be faced even non-medical personnel in recent society.

In addition, a potential benefit that radial pulse palpitation could estimate systolic blood pressure (SBP) has been reported in previous studies^5,6^., For example, after initial evaluation, 325 trauma patients were characterised as having a ‘normal’ (n = 305) or ‘weak’ (n = 20) radial pulse by paramedics, and the difference in SBP between the normal and weak groups was approximately 30 mmHg^5^. Major international guidelines, such as the European Resuscitation Council and American Heart Association, suggest that even health professionals consider the technique of carotid artery palpation to be difficult to learn and maintain^2,7^. However, to the best of our knowledge, no studies in the literature have reported the accuracy of radial artery palpation of non-medical participants. As for breathing, several studies have revealed that the determination of the presence of breathing by a non-medical person had a sensitivity of 80–99%, specificity of 50–74%, and accuracy of 80%^8,9^. However, potential bias due to different sample sizes (n = 48–118)^8,9^, different methods (simulation or video clips)^8^, or different types of participants (non- or medically trained persons)^9^ exists, requiring future investigation.

The use of high-fidelity manikins for advanced life support training is associated with moderate benefits for improved skill performance at course conclusion^10^. High-fidelity simulation training is superior to low-fidelity cardiopulmonary resuscitation (CPR) manikin training in terms of teaching implementation of high-quality CPR for chest compression depth and compression fraction to fourth-year medical students^11^. Short-duration, spaced CPR training on a manikin with real-time visual feedback is effective in improving CPR performance^12^. High-fidelity simulators have been used in previous studies because they allow adjustments of breathing rate, radial pulse rate, and radial pulse strength^9^.

Therefore, this study aimed to determine the accuracy of breathing assessment and radial pulse palpation among 450 non-medical personnel using a high-fidelity simulator.

## Methods

### Experimental design and study participants

This observational cross-sectional study was conducted from August 1, 2016, to August 19, 2018, in Saitama and Tokyo, Japan. In total, 496 primary to high school teachers were included in the study. A questionnaire was administered to the participants to ascertain their medical licence status; age; sex; and whether they had ever requested an ambulance, performed CPR, or attended a CPR training course; and if yes, when. We excluded 43 participants (8.7%) with missing medical qualifications data and 29 participants (5.8%) with medical qualifications. Finally, 424 participants (85.5%) were included in the analyses. The background details of the participants are presented in Table 1.

**Table 1:** Baseline characteristics of the participants.

Baseline characteristics	n = 424
Age (year)—mean (SD)	40 (8.6)
Female sex—n (%)	335 (79.0)
Have an ambulance request judgement experience—n (%)	367 (86.6)
Have a CPR experience—n (%)	64 (15.1)
CPR training n (%)	
No experience	3 (0.7)
3 months	164 (38.7)
4–6 months	19 (4.5)
7 months to 1 year	82 (19.3)
1–3 year	95 (22.4)
> 3 year	58 (13.7)
Missing	3 (0.7)

*CPR* Cardiopulmonary resuscitation.

Before the experiment, all participants were informed about the study's purpose and procedures. After receiving a description of the experiment, they signed an agreement with informed consent to participate in the study. The study was conducted in accordance with the ethical guidelines for medical and health research involving human subjects. This study was approved by the Institutional Review Board of the Nippon Sports Science University (Approval Number 016-H020).

### Equipment

The sensitivity, specificity, and accuracy of breathing and radial pulse assessment were determined using the Resusci Anne Simulator® (Laerdal Medical AS, Stavanger, Norway) and SimPad^®^ (Laerdal Medical AS). The validatory of blood pressure accuracy is ± 4 mmHg^13^. Four scenarios of breathing were defined: ‘no breathing’ [respiratory rate (RR) = 0 breaths per minute (BPM)]; ‘slow’ breathing [RR = 9 BPM]; ‘normal’ breathing [RR = 18 BPM]; and ‘fast’ breathing [RR = 30 BPM]. All respiratory sounds were turned off in the simulator. The simulator can set three levels of radial pulse strength. Ten scenarios of radial pulse were defined: ‘no pulse’ [SBP = 0 mmHg and pulse rate (PR) = 0 bpm]; ‘weak’ pulse [SBP = 80 mmHg]; ‘normal’ pulse [SBP = 120 mmHg]; and ‘strong’ pulse [SBP = 180 mmHg]. Radial pulse rate was categorised as follows: ‘slow’ pulse [PR = 49 bpm]; ‘normal’ pulse [PR = 80 bpm]; and ‘fast’ pulse [PR = 120 bpm]. All participants were asked randomly to judge these breathing and radial pulse scenarios.

### Assessment procedure

The validation was conducted in a double-blind manner for the participants and evaluators. The participants were seated on the right side of the simulator and asked to assess breathing in the simulator for 10 s. A random number was selected on the SimPad, the evaluator signalled the start button, and the SimPad monitor was set to 10 s. The participants started and stopped their assessment at the evaluator's signal. The participants were asked to assess breathing for 10 s and judge the absence or presence of breathing; if breathing was present, they were asked to determine whether the breathing was slow, normal, or fast.

The participants were seated on the right side of the simulator and were asked to assess the normal strength and rate of radial pulse for 10 s. A random number was selected on the SimPad, the evaluator signalled the start button, and the SimPad monitor was set to 10 s. The participants started and stopped their estimation at the evaluator’s signal. They were asked to assess the absence or presence of radial pulse for 10 s; if the radial pulse was present, they were asked to judge whether the strength was weak, normal, or strong, and whether the rate was slow, normal, or fast.

### Outcomes

The primary outcome was the accuracy of the presence or absence of breathing and pulse, and the secondary outcome was the accuracy of breathing rate, pulse rate, and pulse strength.

### Statistical analyses

Age is presented as mean [standard deviation (SD)]. The sensitivity, specificity, and accuracy of breathing and radial pulse assessment were calculated. The relationship among pulse strength, pulse rate, and breathing rate was analysed using Spearman’s correlation coefficient. All statistical analyses were performed using the EZR software (Saitama Medical Center, Jichi Medical University, Saitama)^14^, which is a modified version of R commander designed to add statistical functions frequently used in biostatistics.

### Ethics approval and consent to participate

This study was approved by the Institutional Review Board of the Nippon Sports Science University (Approval Number 016-H020). Before the experiment, all participants were informed about the study’s purpose and procedures. After receiving a description of the experiment, they signed an agreement with informed consent to participate in the study.

## Results

The mean age of the participants was 40 (8.6) years. Among a total of 424 participants, 89 (21%) were men and 335 (79%) were women. A total of 367 (86.6%) participants made the decision to call an ambulance, and 64 (15.1%) participants performed CPR for a patient who experienced CPA, whereas 3 participants (0.7%) never attended CPR training. The last CPR training attendance was attended within 3 months for 164 (38.7%), 4–6 months for 19 (4.5%), within 7 months to 1 year for 82 (19.3%), 1–3 years for 95 (22.4%), and > 3 years for 58 (13.7%) participants, whereas the remaining 3 (0.7%) participants had no answer.

The breathing scenario settings were as follows: 0 BPM, 100 (23.6%); 9 BPM, 122 (28.8%); 18 BPM, 102 (24%); and 30 BPM, 100 (23.6%). The radial pulse scenario settings were as follows: no pulse (SBP 0 mmHg/PR 0 bpm), 45 (10.6%); SBP 80 mmHg/PR 49 bpm, 38 (9%); SBP 80 mmHg/PR 80 bpm, 43 (10.1%); SBP 80 mmHg/PR 120 bpm, 52 (12.3%); SBP 120 mmHg/PR 49 bpm, 34 (8%); SBP 120 mmHg/PR 80 bpm, 41 (9.7%); SBP 120 mmHg/PR 120 bpm, 40 (9.4%); SBP 180 mmHg/PR 49 bpm, 48 (11.3%); SBP 180 mmHg/PR 80 bpm, 41 (9.3%); and SBP 180 mmHg/PR 120 bpm, 42 (9.9%) times (Table 2).

**Table 2:** The number of breathing and radial pulse scenarios.

Breathing scenario	n (%)
RR 0 BPM	100 (23.6)
RR 9 BPM	122 (28.8)
RR 18 BPM	102 (24.0)
RR 30 BPM	100 (23.6)
Radial pulse scenario	
SBP 0 mmHg/PR 0 bpm	45 (10.6)
SBP 80 mmHg/PR 49 bpm	38 (9.0)
SBP 80 mmHg/PR 80 bpm	43 (10.1)
SBP 80 mmHg/PR 120 bpm	52 (12.3)
SBP 120 mmHg/PR 49 bpm	34 (8.0)
SBP 120 mmHg/PR 80 bpm	41 (9.7)
SBP 120 mmHg/PR 120 bpm	40 (9.4)
SBP 180 mmHg/PR 49 bpm	48 (11.3)
SBP 180 mmHg/PR 80 bpm	41 (9.7)
SBP 180 mmHg/PR 120 bpm	42 (9.9)

*RR* respiratory rate, *BPM* breaths per minute, bpm beats per minute, *SBP* systolic blood pressure, *PR* pulse rate.

Four scenarios of breathing were defined: ‘no breathing’, RR = 0 BPM; ‘slow’, RR = 9 BPM; ‘normal’, RR = 18 BPM; and ‘fast’, RR = 30 BPM. All respiratory sounds were turned off in the simulator.

Ten scenarios of radial pulse were defined: ‘no pulse’, SBP = 0 mmHg and PR = 0 bpm; ‘weak’, SBP = 80 mmHg; ‘normal’, SBP = 120 mmHg; and ‘strong’, SBP = 180 mmHg. Radial pulse rate was categorised as follows: ‘slow’, PR = 49 bpm; ‘normal’ PR = 80 bpm; and ‘fast’, PR = 120 bpm.

The presence of breathing was correctly answered by 408 (96.2%) of the 424 participants. For the setting of no breathing, 92 of 100 (92%) participants answered correctly, and for the setting of breathing, 316 of 324 (97.5%) participants answered correctly. The presence of radial pulse was answered correctly by 389 of 424 (91.7%) participants. For the scenario of no radial pulse, 42 of 45 (93.3%) participants answered correctly, and for the scenario of pulse, 347 of 379 (91.6%) participants answered correctly. The sensitivity for checking the presence or absence of breathing and radial pulse was 97.5% and 99.1%, respectively. The specificity was 92% for breathing and 56.8% for a radial pulse. The overall diagnostic accuracy for breathing and radial pulse was 96.2% and 91.7%, respectively (Table 3). The breathing rate was answered correctly by 257 (81.3%) of 316 participants. For the slow breathing setting, 102 (87.2%) of 117 participants answered correctly; for the normal breathing setting, 77 (77%) of 100 participants answered correctly; and for the fast breathing setting, 78 (78.8%) of 99 participants answered correctly.

**Table 3:** Measures of sensitivity, specificity, and accuracy of breathing and radial pulse.

	Sensitivity (%)	Specificity (%)	Accuracy (%)
Breathing (95% CI)	97.5^a^ (95.2–98.9)	92.0^c^ (84.8–96.5)	96.2 (93.9–97.8)
Radial pulse (95% CI)	99.1^b^ (97.5–99.8)	56.8^d^ (44.7–68.2)	91.7 (88.7–94.2)

^a^Proportion of times the participants accurately stated that breathing was 316 of 324.

^b^Proportion of times the participants accurately stated that radial pulse was 347 of 379.

^c^Proportion of times the participants accurately stated that no breathing was 92 of 100.

^d^Proportion of times the participants accurately stated that no radial pulse was 42 of 45.

*CI* confidence interval.

The sensitivity of breathing rate assessment was as follows: slow, 87.2%; normal, 72.6%; and fast, 83.9%. The specificity was 92.5%, 89%, and 90.6%, respectively. The overall diagnostic accuracies were 90.5%, 83.5%, and 88.6%, respectively. The results were correlated, with a Spearman’s correlation coefficient of 0.813 (*P* < 0.001) (Table 4). Radial pulse strength was correctly answered by 148 (42.7%) of the 347 participants. For the weak radial pulse setting, 42 (35.6%) of 118 participants answered correctly; for the normal radial pulse setting, 57 (53.3%) of 107 participants answered correctly; and for the strong radial pulse setting, 49 (40.2%) of 122 participants answered correctly. The sensitivity of radial pulse strength assessment was as follows: weak, 56%; normal, 32.8%; and strong, 50%. The specificity was 72.1%, 71.1%, and 70.7%, respectively. The overall diagnostic accuracies were 68.6%, 51.9%, and 64.8%, respectively. The results were weak but significant correlation with a Spearman's coefficient of 0.288 (*P* < 0.001) (Table 5). Radial pulse rate was answered correctly by 228 (65.7%) of the 347 participants. For the slow radial pulse setting, 36 of 104 (34.6%) answered correctly; for the normal radial pulse setting, 79 of 118 (66.9%) answered correctly; and for the fast radial pulse setting, 113 of 125 (90.4%) answered correctly. The sensitivity of radial pulse rate assessment was as follows: slow, 83.7%; normal, 52.3%; and fast, 73.9%. The specificity was 77.6%, 80.1%, and 93.8%, respectively. The overall diagnostic accuracies were 78.4%, 68%, and 85%, respectively. The results were correlated, with a Spearman's correlation coefficient of 0.719 (*P* < 0.001) (Table 6).

**Table 4:** Measures of sensitivity, specificity, and accuracy of speed of breathing.

	Sensitivity (%)	Specificity (%)	Accuracy (%)
Overall (95% CI)	81.3 (76.6–85.5)	90.7 (88.1–92.8)	87.6 (85.3–89.6)
Slow (95% CI)	87.2 (79.7–92.6)	92.5 (87.9–95.7)	90.5 (86.7–93.5)
Normal (95% CI)	72.6 (63.1–80.9)	89.0 (84.0–92.9)	83.5 (79.0–87.5)
Fast (95% CI)	83.9 (74.8–90.7)	90.6 (86.0–94.1)	88.6 (84.6–91.9)

*RR* respiratory rate, *BPM* breaths per minute.

We defined four scenarios of breathing: ‘no breathing’, RR = 0 BPM; ‘slow’, RR = 9 BPM; ‘normal’, RR = 18 BPM; and ‘fast’, RR = 30 BPM.

RR 0 BPM data were excluded (n = 100).

Incorrect answers regarging the presence of breathing were excluded (n = 8).

*CI* confidence interval.

**Table 5:** Measures of sensitivity, specificity, and accuracy of radial pulse strength.

	Sensitivity (%)	Specificity (%)	Accuracy (%)
Overall (95% CI)	42.7 (37.4–48.0)	71.3 (67.8–74.7)	61.8 (58.7–64.7)
Weak (95% CI)	56.0 (44.1–67.5)	72.1 (66.3–77.3)	68.6 (63.4–73.4)
Normal (95% CI)	32.8 (25.8–40.3)	71.1 (63.7–77.7)	51.9 (46.5–57.2)
Strong (95% CI)	50.0 (39.7–60.3)	70.7 (64.6–76.3)	64.8 (59.6–69.9)

Spearman’s rank correlation coefficient: rs = 0.288, P < 0.001.

*SBP* systolic blood pressure, *PR* pulse rate, *bpm* beats per minute.

We defined ‘weak’, SBP = 80 mmHg; ‘normal’, SBP = 120 mmHg; and ‘strong’, SBP = 180 mmHg.

SPB 0 mmHg and PR 0 bpm data were excluded (n = 45).

Incorrect answers regarging the presence of radial pulse were excluded (n = 32).

*CI* confidence interval.

**Table 6:** Measures of sensitivity, specificity, and accuracy of speed of radial pulse.

	Sensitivity (%)	Specificity (%)	Accuracy (%)
Overall (95% CI)	65.7% (60.4–70.7)	82.9 (79.8–85.6)	77.1 (74.5–79.7)
Slow (95% CI)	83.7 (69.3–93.2)	77.6 (72.5–82.2)	78.4 (73.7–82.6)
Normal (95% CI)	52.3 (44.0–60.5)	80.1 (73.8–85.5)	68.0 (62.8–72.9)
Fast (95% CI)	73.9 (66.1–80.6)	93.8 (89.4–96.8)	85.0 (80.8–88.6)

Spearman’s rank correlation coefficient: rs = 0.719, P < 0.001.

*SBP* systolic blood pressure, *PR* pulse rate, *bpm* beats per minute, *CI* confidence interval.

Radial pulse rate was categorised as follows: ‘slow’, PR = 49 bpm; ‘normal’ PR = 80 bpm; and ‘fast’, PR = 120 bpm.

SPB 0 mmHg and PR 0 bpm data were excluded (n = 45).

Incorrect answers regarding the presence of radial pulse were excluded (n = 32).

## Discussion

In this study, the accuracy of breathing assessment and radial pulse palpation was examined among 450 non-medical persons using a high-fidelity simulator. The evaluation for the presence of breathing had a 96.2% correct response rate, with > 90% sensitivity, specificity, and accuracy. The assessment of the presence of radial pulse had a correct response rate of 91.7%, with a sensitivity of 99%, accuracy of 91.7%, and specificity of 56.8%.

For the detection of the presence of breathing, the sensitivity, specificity, and accuracy were all > 90%. These results were higher than those reported in previous studies. For example, Perkins et al.^8^ examined 48 s-year medical students to assess the presence of breathing in six videos and reported 88% sensitivity, 74% specificity, and 81% accuracy. The videos contained three different no breathing scenarios, two of which were agonal breathing. Participant background and assessment method may have caused the high correct answer rate, sensitivity, specificity, and accuracy.

The sensitivity and accuracy for radial pulse detection exceeded 90%, but the specificity was only 56%. Previous studies have reported carotid artery palpation as a method for detecting cardiac arrest in children; both civil rescuers and medical personnel have reported difficulty in learning and maintaining techniques to identify the carotid artery^8,15–19^. Another report measured the time required by anaesthesiologists to confirm the carotid artery and radial pulse in 554 anaesthetised patients. Radial pulse was identified most rapidly [98% in 5 s and > 99% in 10 s], whereas the carotid artery was not easily identified [81.5% in 5 s and 96.7% in 10 s]^20^. In the present study, we performed radial pulse palpation instead of carotid artery palpation. The sensitivity (99.1% vs. 98.9%), specificity (56.8% vs. 48.9%), and accuracy (96.2% vs. 78.9%) of radial artery palpation in the present study were comparable with those in a previous study^9^. However, the previous study enrolled 118 health care professionals and university students who had taken basic life support lessons; thus, as the participants were more experienced, selection bias was possible. In the present study, 424 schoolteachers and staff evaluated the scenarios only once and in a double-blind manner, leading to minimum bias in the results.

The correlation coefficient for breathing rate was 0.81 (sensitivity, 72–87%; specificity, 89–93%; and accuracy, 84–91%), indicating a strong correlation. In a previous study, the diagnostic accuracy of 48 medical students in judging normal or abnormal/absent breathing was also poor (sensitivity, 60%; specificity, 75%; and accuracy, 72%)^8^. In the present study, the sensitivity, specificity, and accuracy were higher probably because absent breathing was not included. For slow and fast breathing, the accuracy was approximately 90%, and there was a strong correlation between the rates. The breathing rate, which was approximately 90% accurate, had a strong correlation with the assessment, indicating that the participants could accurately estimate their breathing rate.

The correct response rate for radial pulse strength was 42.7% and the radial pulse rate was 65.7%. For radial pulse strength, the correlation with the assessment was weak, and the overall sensitivity, specificity, and accuracy were low, especially for normal strength, with 33% sensitivity, 71% specificity, and 52% accuracy. The radial pulse rate showed a strong correlation with the assessment, showing the lowest sensitivity and accuracy in the normal scenario, and the lowest specificity in the slow scenario. For breathing rate, the participants had difficulty judging normal breathing. In contrast, the sensitivity and specificity for breathing rate were 74% and 94%, respectively, indicating that the participants judged fast or not fast breathing. To the best of our knowledge, this is the first study on the sensitivity, specificity, and accuracy of radial artery palpation by non-medical persons.

The strength of this study is the large sample size of 450, which is four times larger than that of previous studies^8,9^. In addition, the participants were limited to schoolteachers only, thereby minimising variations in their backgrounds. Further, 91% of participants were successfully completed our primary assessment.

This study has several limitations. As this was an observational and cross-sectional study, only associations were observed and not causal relationships. Moreover, the accuracy of the assessments still needs to be verified with that of actual patients. Furthermore, as the simulator used was designed for adults, it is unclear whether the results of this study can be applied to children.

In conclusion, our study suggested that non-medical persons would show high accuracy in detecting and measuring respiration and radial pulse but were not able to accurately determine radial pulse strength in the early recognition of CPA.

## Data Availability

The data that support the findings of this study are available from the corresponding author, K.S., upon reasonable request.

## Abbreviations

SBP: Systolic blood pressure
CPA: Cardiopulmonary arrest
CPR: Cardiopulmonary resuscitation
BPM: Breaths per minute
bpm: beats per minute
PR: Pulse rate
RR: Respiratory rate
